# Perceptions of Health and Wellbeing Among Employees in a Work Integration Social Enterprise in Sweden

**DOI:** 10.5334/aogh.4065

**Published:** 2023-05-12

**Authors:** Gloria Macassa, Cormac McGrath, Michael J. Roy, Frida Stål, Anne Sofie Hiswåls, Mamunur Rashid, Ulf Karlsson, Rooney Olsson, Jose Pedro Silva, Stig Vinberg, Anneli Marttila

**Affiliations:** 1 Department of Public Health and Sports Science, Faculty of Occupational and Health Sciences, University of Gävle, Kungsbacksvägen 47, 80176 Gävle, Sweden; 2 EPIUnit–Instituto de Saude Publica, Universidade do Porto, Rua das Taipas 135, 4050-600 Porto, Portugal; 3 Laboratório para a Investigação Integrativa e Translacional em Saúde Populacional (ITR), Rua das Taipas, nº 135, 4050-600 Porto, Portugal; 4 Department of Education, Stockholm University, Frescativägen 54, 10691 Stockholm, Sweden; 5 Yunus Centre for Social Business and Health, Glasgow Caledonian University, M201, George Moore Building, Glasgow G4 0BA, United Kingdom; 6 Coompanion Gävleborg, Drottninggatan 18, 803 20 Gävle, Sweden; 7 Instituto de Sociologia da Universidade do Porto, Faculdade de Letras da Universidade do Porto, Via Panorâmica, 4150-564, Porto, Portugal; 8 Department of Health Sciences, Faculty of Humanities, Mid Sweden University, Holmgatan 10, 851 70 Sundsvall, Sweden

**Keywords:** work integration social enterprise, Sweden, employee health and wellbeing

## Abstract

**Background:**

Work Integration Social Enterprises (WISEs) constitute an important vehicle for providing employment opportunities for disadvantaged groups.

**Objective:**

The goal of this qualitative case study is to explore perceptions of health and wellbeing among employees working in a WISE located in the Gävleborg region, in east central Sweden.

**Methods:**

Data were gathered using 16 in-depth, semi-structured interviews with the social enterprise employees.

**Results:**

Findings were categorized into three main categories: the importance of financial independence and societal benefits; team spirit and a sense of belonging; and improved quality of life and wellbeing.

**Conclusion:**

The participants perceived that working in the WISE gave them a feeling of freedom and increased their self-esteem because of the possibility to earn an income. Also, they were satisfied with their job (e.g., with regard to work quality and flexibility) and believed that their work contributed to society. Moreover, through working in a WISE, the participants felt a sense of belonging and togetherness through interaction with co-workers and managers, and an improved quality of life for themselves and their families.

## Introduction

Social enterprises (SEs) are businesses whose primary goal is to achieve a social impact. Rather than generating profits for owners and shareholders they use their surpluses mainly to achieve social goals. Social enterprises are managed in an accountable, transparent, and innovative way, in particular by involving workers, customers, and stakeholders affected by their business activity [1]. Through addressing local vulnerabilities that are known to favour or harm health, SEs are considered to improve public health across disadvantaged segments of the population (e.g., through provision of employment and training). In addition, they can also provide an array of programmes, products and services to populations experiencing inequalities in health [1,2,3,4,5].

In recent decades, various studies have pointed out that SEs have the potential to enhance the public health of socially disadvantaged people given that governmental efforts alone may not be enough to address the root causes of inequalities in health [5,6,7]. Empirical evidence of the link between SE activities and public health has come from an array of studies carried out in different contexts and using both qualitative and quantitative designs targeting disadvantaged groups [8,9,10,11,12,13,14,15]. For instance, using theories related to capability, social capital, and space of wellbeing, some studies have shown that SEs enhance health and wellbeing [4,15]. However, some argue that many of the studies showing evidence of how SEs relate to public health and wellbeing have relied on small numbers of cases and on interview data [2,16]. Furthermore, there are those who state that most of the available empirical evidence has not taken into consideration the capacities of SEs, which may directly or indirectly affect their effectiveness in supporting socially vulnerable population groups [2].

In a study carried out in Korea using multilevel modelling, government-certified SEs were found to be associated with positive self-rated health among low-income residents, but some SEs such as those at an early stage in their development, with insufficient profitability and weak corporate governance, tended to show mixed results [2]. Other empirical evidence suggests that SEs are effective in reducing marginalization and stigmatization in disadvantaged groups and can build health assets, social capital, quality social networks, and relationships – as well as provide stable employment; give access to blue and green spaces; and lead to improved health behaviours [6,17,18]. Empirical evidence of effective SEs has been found across different societal groups, such as unemployed people, young adults, older people, people with mental illnesses, asylum seekers, and refugees [16,19,20,21,22]. A study by McKinnon et al. [24] found that SEs implicitly and explicitly shape their operations around enhancing wellbeing (e.g., through on-site activities, relationships forged with clients and stakeholders, and market relations).

Studies carried out in various fields (e.g., economics or sociology) addressing the impact of SEs on wellbeing have evolved to acknowledge the role that geography and context might have on the observed evidence [25]. Roy et al. argue that future research needs to take this issue seriously, particularly given the role that SEs play in potentially modifying the determinants of health, and that close attention needs to be given to the complexities of navigating and appropriately considering the mitigating role of social and economic policies [26]. Also, Suchowerska and colleagues raise the point that much of current research has focused on the transactional organizational features that are most apparent in daily life, including interpersonal relationships and the allocation of tasks, thus potentially neglecting transformational features such as organizational strategy and leadership [27].

In relation to Work Integration Social Enterprises (WISEs), which are the target of this study, evidence shows that there is an association between their activities and improved wellbeing among users [23,28]. For instance, Barraket et al. recently reported that, in Australia, WISEs can increase young people’s access to employment and/or employability, improve their self-reported mental health and wellbeing, and positively influence healthier behaviours, including healthy eating, reduced smoking, and reduced drug use [23]. In addition, WISEs were reported to contribute to the improvement of young people’s confidence and social skills in professional and personal contexts, as well as fostering positive new relationships and connections [23]. Also, WISE strategies and organizational processes in many cases influence health outcomes through having an impact on social determinants of health, such as employment and income, in turn offering a sense of purpose, structure, and opportunities for social cohesion and having a direct benefit on mental health (e.g., through improved self-esteem and social connection as well as reduced anxiety and depression) [28]. A recent study carried out in Spain found that different groups of employees created their own narrative of working in WISEs based on experiences of meaningful work; employees with no disadvantage experienced improvements in eudaemonic wellbeing (meaning and purpose) while those in disadvantage reported improvements in hedonic (happiness) wellbeing [29].

In Sweden, SE (and WISE) ecosystems differ slightly from those of other countries [30] as these enterprises are strictly embedded within the context of a strong welfare system [31]. The country has a long and historical tradition of social engagement in social welfare. In the pre-welfare state, social initiatives were predominantly designed to target socially disadvantaged groups in a historically poor society [31,32]. According to Wijikström and Lundström, this was carried out by popular mass movements (e.g., *Folkrörelser* and *Folkhem*), democratically structured membership organizations that combined social services with advocacy in society [33]. Then, in the late 19th and in the 20th century, with the firm establishment of the social democratic model of governance [34], the Swedish social welfare system dominated the provision of welfare services such as health care, child care, elderly care, and social care for people with special needs. However, during this same period, elderly and social care started to become deregulated and sometimes run by private providers as a complement to the public system. These initiatives were mainly funded through fundraising activities, membership fees, and public grants (rather than sales) [31,35]. Currently, SEs (including WISEs) are flourishing in tandem with the social welfare system, although there are still no specific legal structures regulating them [36,37]. These SEs (i.e., WISEs, non-profit SEs, social-purpose businesses, and societal entrepreneurships) combine social missions with some kind of business activity. However, WISEs (as explored in the present case study) have by far the strongest framework [36].

According to Defourny et al., [30] in Nordic countries including Sweden, although the rise of SEs (including WISEs) was anchored in a strong civil society and cooperative tradition, and has been driven mainly by grassroots entrepreneurial initiatives, ‘the opportunity structure for the emergence and further developments of [SEs] is to a great extent delimited by the framework of welfare policies’ [30]. They further argue that in countries such as Sweden there is a tension between policy discourse at the local level, emphasizing social innovation, civil society initiatives, collaborative governance – and the policy instruments that, at the central level of government, are still inspired by new public management (NPM) principles [38], but remain characterized by their market orientation and reliance on quasi-market mechanisms [30]. Thus, SEs are exposed to different forms of pressures which might force them to lose their organizational specificities as well as their real capacity to innovate.

Today, Sweden’s SE sector is still fairly small and lacks a specific legal framework, and the organizations in this sector use legal forms such as economic associations (*ekonomiska föreningar*) or non-profit associations (*ideella föreningar*) [33]. Furthermore, the SE concept is most commonly associated with WISEs, which, as mentioned above, aim to integrate people into society and working life through focusing on groups such as migrants and unemployed people, and providing rehabilitation and work experience to those with a history of drug and alcohol abuse.

The relationship between the activities of WISEs and individual and community wellbeing has been explored using various theoretical frameworks. This study explores the experiences and perceptions of the effect of WISEs on health and wellbeing through the lens of a conceptual framework developed by Roy et al. [3,7]. It proposes that, as upstream interventions, SEs have an impact on the social determinants of health, that is, ‘the conditions in which people are born, grow [up], live, work and age’, which are ‘shaped by the distribution of money, power and resources at global, national and local levels’ [39,40]. According to the framework, SEs can intervene directly (through trading or service delivery) or indirectly (through trading activities that generate profits reinvested in assets), and ultimately and in the long term can improve the health and wellbeing of beneficiaries. The health ‘assets’ that can be affected by SEs include strengthening social networks and relationships, trust and safety, emotional support, good work, social functioning, emotional wellbeing, and lifestyle [3,7]. In the specific case of WISEs, the framework suggests that they provide meaningful work, safe and supportive environments, improved knowledge and skills, and expansion of social networks. They also contribute to build trust and cooperation, improving physical health through a safe and supportive work environment, and mental wellbeing through strengthening confidence and feelings of empowerment, a sense of personal pride and dignity, self-esteem, and self-worth [3,7]. Furthermore, drawing on a systematic review, the framework suggests that WISEs can improve satisfaction with life [3,7].

In recent years, and in the context of the United Nations (UN) Sustainable Development Goals (SDGs) and UN Agenda 2030, Sweden has stepped up its efforts towards a better understanding of the activities and processes carried out by SEs. For instance, in 2018 the Swedish government launched a new strategy for SEs, social entrepreneurship and social innovation [41]. Taking a challenge-driven approach, the strategy aims to strengthen the development of SEs so that they can participate in solving societal challenges and contribute to sustainable development. In tandem with the strategy, the government launched a three-year mission, investing 20 million SEK (1.8 million EUR) per year, respectively, in the Swedish Agency for Economic and Regional Growth and the Swedish Innovation Agency, to promote a range of SE activities. These include advisory activities, competence development, support for business development and knowledge dissemination, support to incubators, and development of impact assessment measures [41]. Although there is considerable interest in, and support for, the activities of the country’s SEs, no previous research has been undertaken in Sweden that has related the work of these entities to health and wellbeing. Therefore, to fill this knowledge (and policy) gap, this study aimed to explore perceptions of health and wellbeing among employees working in a WISE located in Gävleborg, a region in east central Sweden.

## Methods

### Study Design and Sample

This study applied a qualitative case study approach, using in-depth, semi-structured interviews [42,43] to intensively focus on the wide and varied activities of one WISE purposively selected for being especially ‘data-rich’. According to Yin [44], a case study is an enquiry that investigates a contemporary phenomenon in-depth and within its real-life context, especially when the boundaries between the phenomenon and the context are not clearly evident. Furthermore, Yin argues that, for answering the ‘how’ and ‘why’ question, qualitative methodology is the suitable choice for the exploration of participants’ experiences and for obtaining deeper, meaningful insights into real-life situations [44].

The WISE the present case study is based on is located in east central Sweden, in the region of Gävleborg, which has a population of approximately 290 000 residents living in ten municipalities [45] and historically has been linked to the steel, forestry, and engineering industries. However, in recent years, private services have become the fastest growing sector (including environmental technology, information technology (IT) businesses, tourism, and the hospitality sector), employing the majority of residents [46]. The region is characterized by low levels of education and high unemployment rates that are above the national average, especially among young adults [47]. Furthermore, data suggest that the region also has a higher proportion of individuals with low disposable income, as well as a high proportion of people categorized as being in vulnerability (e.g., immigrants, single elderly people, single women, single parents, disabled people, unemployed people, and people on long-term sick leave). In addition, compared with the national average, the region has high levels of self-rated ill health and lower than average life expectancy [47]. In recent years, and following the establishment of the national policies, the region has doubled its efforts to establish a regional agency for SEs, to integrate the concept of SEs into existing policies, develop a strategy for financing, and create SE hubs and incubators [48].

Founded in 2017, the WISE in question is a self-financed cooperative. Its vision is that all people are needed and that every individual who finds his or her way becomes a resource. The main goal is to create adapted jobs and provide work training for individuals in long-term unemployment or on long-term sick leave and people with disabilities. The WISE provides services such as home and office cleaning, gardening, and snow clearing, as well as rehabilitation, provision of school lunches, and small coffee shop services in some local public schools. The organization has a total of 40 employees, five of whom are managers with the responsibility to run day-to-day activities. For this study, a total of 16 employees, twelve women and four men, completed the interviews and were included in the study. The remaining employees declined to participate in the study. The demographic characteristics of the study participants are presented in Table 1.

**Table 1:** Demographic characteristics of the study participants in the Social Enterprise, East Central Sweden.


	N = 16

**Sex**	

Male	4

Female	12

**Age (y)**	

<29	2

30–39	6

≥40	8

**Education (y)**	

<12	16

≥12	0

**Length of employment in the SE (months)**	

<12	1

12–23	2

24–47	6

≥48	7

**Country of birth**	

Sweden	16

Other	0


### Data Collection and Procedure

To gain an understanding of participants’ perceptions of working in a WISE and potential consequences to their health and wellbeing, the research team developed a semi-structured interview guide with open-ended questions. The original interview guide was written in English, translated into Swedish, and back-translated into English for accuracy. The interview guide contained background (e.g., sociodemographic) questions, and questions related to experiences of working in the WISE and perceptions of job satisfaction, health, and wellbeing (see Table 2).

**Table 2:** Interview guide.


TOPICS OF DISCUSSION FOR THE INTERVIEW

**1.** Can you tell us about your experience of working for your social enterprise?

**2.** How does working here compare to other organizations’ you have worked for?

**3.** Can you tell us how your job is?

**4.** Do you feel that working here affects your health and well-being?

**5.** In terms of health and well-being, have you noticed any differences when working for a social enterprise?

**6.** How do you feel about the social enterprise commitment to reinvest any profits back into the business?

**7.** What do you think about working in your social enterprise?


The recruitment of employees was conducted in two steps. First the research team had a meeting with the chief manager of the WISE to explain the aim of the study and ask permission to interview the employees. After contacting the manager, we invited all employees to participate; 16 accepted the invitation.

The interviews were conducted face-to- face between March and August 2021 and the interview time ranged from 20 to 60 (average 40) minutes. All interviews took place in a secluded and undisturbed room. Prior to the interviews all participants were informed about the study objectives and (after assurances of confidentiality) asked to give their verbal consent.

### Data Analysis

Qualitative content analysis as proposed by Graneheim and Lundman [49] was used to analyse the transcripts of the in-depth, semi-structured interviews. The steps required in content analysis include identifying meaning units in the material and summarizing the information into condensed meaning units and coding these, building categories from the codes, and making analytical interpretive connections between the categories [50,51]. In the analysis, the entire interview transcript was considered the unit of analysis, as suggested by Graneheim and Lundman [49]; thus, the focus was on the manifest content.

After the abovementioned steps, the transcribed data were read through repeatedly to make sense of them and achieve immersion. Furthermore, the meaning units reflecting key thoughts were highlighted. The meaning units were assigned headings and were analysed and matched to codes (generated during the initial stage of the analysis). Moreover, the entire transcribed text was systematically coded to cover different aspects of the content in the material to relate it to the study’s main purpose. The codes that shared similarities were grouped into several broader categories to form meaningful clusters.

The primary analysis was performed by researchers (FS and GM), in discussion with the research team. During the process of sorting and abstraction, the two researchers repeatedly compared the categories for similarities and differences, building a hierarchy of codes, categories, and sub-categories, which were further discussed with the rest of the research team. All analyses were carried out manually.

### Ethical Considerations

The research protocol of the study was approved by the Swedish Ethical Review Authority (approval No. 2020-06521). Furthermore, all participants received an explanation of the purpose of the study and provided verbal informed consent before their interview. Participants were also informed about their right to withdraw from the study at any time, before or during the interview. Furthermore, the WISE was anonymized to ensure that it is not identifiable.

## Results

The findings are presented as three main categories capturing employees’ experiences and perceptions of working in a WISE: (1) the importance of financial independence and societal benefits; (2) team spirit and a sense of belonging; and (3) improved quality of life and wellbeing. We also provide samples of anonymized quotes to illustrate the findings.

**1. The importance of financial independence and societal benefits**

The participants in the studied WISE talked about achieving financial security and freedom, as well as being able to contribute to the wider society. Regarding financial security, WISE employees talked about a sense of freedom related to having a proper income and not needing to depend on relatives or the welfare system. One respondent reflected,

So I never had it better … to have freedom. I used to cost a lot of tax money but now have an income. (Interviewee K)

Moreover, employees said that the WISE gave them employment, which in turn provided them with freedom and self-esteem. They also pointed out that working in a WISE was better than working for other types of organizations they had been with in the past; also, that WISE allowed flexibility in terms of working hours. One employee explained,

As I said before, people in my situation can get the chance to get a job that gives financial freedom, and are able to make money themselves. (Interviewee N)

Participants also felt that the work they did in the WISE was very important, not only for themselves, but also for the entire organization. One employee talked about this experience of working in the WISE:

You feel that, oh, what do I do? I actually do things for myself and for everyone else. If you do not do a good job, it affects everyone else. (Interviewee U)

Regarding the WISE’s contribution to society, the participants, all employees of the WISE, perceived that the organization was important because it provided employment for people who had difficulties in finding work, thus reducing societal costs related to unemployment benefits. One employee reflected on how the studied WISE contributed to society:

With work, you can control yourself. You get to work and earn your own money. I think that’s the coolest thing. In the past, you were always told that you only cost the society money. But then when you got that salary, you’re happy and you’ve grown and you’re able to say, ‘I’ve earned this myself.’ (Interviewee L)

Another WISE employee talked about how their clients in the community appreciated their work:

It feels good, and you get appreciation. We usually clean stairs as well and then you get a lot of positive feedback from those who live there. (Interviewee I)

**2. Team spirit and a sense of belonging**

The interviewees discussed the team spirit in the WISE they worked for. The team spirit among the employees involved acceptance of each other, a sense of belonging, and togetherness. Regarding acceptance of each other, the interviewees talked about being accepted in the WISE as they were, irrespective of their previous individual circumstances:

This is a place for me. I can be myself here. Here I get to be who I am. When I was younger, I was ashamed of my disability. Here I started to learn that it’s okay to be who I am as there are other people here with different limitations. (Interviewee N)

The interviewees also experienced feelings of togetherness when working in the WISE:

It’s nice to sit down and talk to co-workers about what they’ve been up to for work and then there is general talk about what you’re interested in doing when you’re at home. (Interviewee J)

In addition, participants felt a sense of belonging through their interaction with colleagues and managers. There was the perception that they were welcome and that their colleagues and managers accepted them as if they were family. One interviewee commented that it was much better working for a WISE compared with previous workplaces:

I think I got off to a very good start here. Considering that I have worked in other places and been on sick leave, which has made me feel that I’m not needed. [And] that I cannot manage a job; and that I have withdrawn a little. Poor self-esteem … stuff like that. They make me feel very good here; I’m satisfied, I look to what I’ve done here, what I can do, and they let me know how good I am. I have grown in my work here, so it’s great fun. (Interviewee U)

**3. Improved quality of life and wellbeing**

The interviewees felt that their work provided them with some semblance of relaxation, work flexibility (e.g., working hours), and improved quality of life and wellbeing.

Had I come to a normal workplace [and not a WISE], it would never have worked for me. Here, you start with the introduction and slowly you get into everything. This work was my salvation in every way. So, this is my first job, yes, my first real job. So, I’m completely satisfied of course. (Interviewee L)

Participants also explained that working for a WISE made them feel safe with co-workers and that having employment gave them more self-esteem as it enabled them to have better control over their lives.

Those I work with and the boss have given me a chance to be who I am. The organization has made me who I am. To dare [to be yourself]! (Interviewee U)

The interviewees also expressed that working in the WISE improved their quality of life and wellbeing. Their previous circumstances had often caused anxiety, depression, and other social problems. Having work and an income facilitated social contacts both at the workplace and in the private sphere. The participants shared how working for a WISE provided an income and it even helped improve the wellbeing of their own children. One interviewee explained:

When I get home from work, I feel that I can do my chores at home. And it means a lot to me. Before, I was very tired, exhausted; I couldn’t stand anything and just lay down. I had anxiety and was depressed. Now I feel much better, I am happy, and have energy left when I get home. (Interviewee A)

### Discussion

The results of this case study suggest that working in a WISE provided the participants with a sense of safety, freedom, and improved self-esteem and that their work contributed to the community and wider society. They also perceived that working in a WISE gave them work quality and job satisfaction: not only did they have flexibility in working hours, but also they felt they could help others; they had a sense of belonging and togetherness through interaction with co-workers and managers. Furthermore, the participants saw employment as an enabler of improved quality of life and wellbeing for both themselves and their families.

Although the results support previous research [4,14,15,52], they provide additional knowledge about how WISEs are affecting people’s wellbeing in the context of a Nordic welfare state where most SEs do not experience a high degree of autonomy [53]. In Sweden, the marketization of welfare services opens up market opportunities but at the same time it increases the competition from the for-profit sector [30]. The WISE in this study was not financed by the national or regional government or municipality, and revenue came solely from selling services to a variety of stakeholders (e.g., municipal organizations, private companies, coffee shops).

As already indicated above, and regardless of the contextual differences with other developed countries, our results resemble those found elsewhere where the impact of WISEs has been studied in relation to the health and wellbeing of beneficiaries [8,9,10,11,12,13,14,15,54].

With regard to the perception of financial security and freedom, this related to the fact that the interviewees had the possibility to have an own income, which freed them from dependence on the social welfare system (e.g., social benefits or unemployment benefits) or relatives. In this case study, employees had previously been in a situation of long-term unemployment (e.g., they had lost their previous employment or had a disability), meaning that they were experiencing exclusion from the labour market as well as experiencing the constant stress of looking for employment. In Sweden, to receive work benefits, unemployed individuals need to prove that they are actively looking for work [55]. This is echoed to some degree in the findings in McKinnon et al.’s study about WISEs in Australia, in which they reported that some participants expressed relief at no longer needing to deal with the pressure from welfare services to seek work [54].

Regarding the participants’ perceived sense of belonging and togetherness in the workplace, this was reflected in the support they received from co-workers and managers as they performed their work duties. It is worth mentioning that they also pointed out that they learned their new work without much pressure and stress and that they were able to socialize and establish a social network with co-workers. Similarly, Joyce et al. [28] found that employees they interviewed felt a strong sense of connection to other people employed in the organization. In a study carried out in Manchester, UK, employees working in an SE perceived that they had adequate control over their jobs, as well as flexibility and support, and that they were involved in decision making and had received appropriate training and development, all of which contributed to job satisfaction [52]. However, the same study in Manchester found that despite the above positive experiences, employees also reported work-life imbalance because of long working hours [52].

As reported, the participants in this study mentioned that working for a WISE improved their quality of life and overall wellbeing. This perception was linked to the fact that, for some, having employment and a proper income lifted them from a combination of relative poverty, distress and depression associated with unemployment and social exclusion. Work also brought increased self-esteem, social networks, and job satisfaction, all of which are linked to feelings of improved wellbeing. Similar findings were reported in a study from Croatia where the interviewed employees felt improvements in their quality of life as a result of increased intrinsic motivation and the perception that their job was valued after they started to work in the studied SE [56]. Investigating Australian WISEs, McKinnon et al. argued that there were different types of wellbeing that could be perceived by beneficiaries. These ranged from material wellbeing (e.g., earning an income to support oneself), social wellbeing (e.g., relationships with co-workers and the ability to meet the demands of the workplace) and physical wellbeing (e.g., skills development, support in learning skills to live healthy lives for greater physical wellbeing) [54]. Likewise, Ho and Chan’s study reported that workers in WISEs perceived improvements in their psychosocial wellbeing because of having an income as well as expanding their networks [57]. Moreover, a study from the UK found that interviewed workers at an SE viewed improvements in their health and wellbeing as a direct and positive effect of working for an SE. Interviewees directly linked the high levels of support and flexibility experienced in the workplace with their increased wellbeing [52]. We can conclude that participants in the present study experienced improved psychological (e.g., reduced stress and anxiety) and social wellbeing (e.g., increased social networks inside and outside the workplace).

Our participants were very positive about their experiences of working in a WISE, which may reflect how precarious and vulnerable their situation must have been before they found employment. Nevertheless, there is evidence elsewhere of WISEs employing people with disabilities (a group also included in our case study), with contrasting results. Efimov et al., for example, reported that, in terms of work content, employees perceived that they had repetitive tasks and that taking responsibility at work caused them stress. This was specifically challenging for employees who were involved in cleaning activities. Also, in that study and with regard to work organization, employees felt that they had a high workload (because of staff shortages), that there was an unfair distribution of work and that collaboration in their team was challenging [58]. Further, according to participants in that study, social relations in that WISE were demanding because of other issues, among them gossip, unsolicited interference, or supervisors putting pressure on them [58].

Overall, the findings obtained from the interviews with employees in the current case study are in line with the pathways of how WISEs can influence the health and wellbeing of their beneficiaries, suggested in Roy et al.’s framework.^3^ For instance, participants who worked in the organization examined in this case study perceived that they had meaningful work that gave them safety, as well as a supporting environment for learning new skills that improved their quality of life.

### Limitations and Strengths

The results of this case study need to be interpreted with caution because of several limitations. Firstly, they are based on a single case study as well a small sample of employees; therefore, they cannot be generalized to WISEs across the entire region of Gävleborg or Sweden as a whole. In addition, the majority of participants had been long-term unemployed, which may have introduced a bias to the results (i.e., eliciting only positive responses). Further studies should attempt to use bigger samples as well as mixed methodology to enable generalizability of the results. However, because this is an explorative qualitative study intended to give an understanding of employees in WISEs, our view is that the findings are reasonably sound. Also, we did not directly ask the participants about their perceptions of the impact of the WISE leadership on their wellbeing.

Nevertheless, the study also has strengths. It is the first ever study of its kind conducted in Sweden to assess perceptions of health and wellbeing among employees in any SE in general, including WISEs. In addition, the study used a social determinants lens indicating the importance of WISEs as a vehicle to improve health equity. Overall, the contribution of this study, which is first in the context of Sweden, is that WISEs appear to make a difference in beneficiaries’ quality of life and wellbeing, even in a context that somewhat limits the degree of autonomy of social enterprises because of the dominance of the provision of services by the social welfare system.

### Implications for Public Health Practice

The findings of this study have important practical implications. Firstly, the employment of people by WISEs in situations of marginalization (e.g., being long-term unemployed, being disabled, or being an immigrant) needs to be supported by government to ensure that these groups feel included in the welfare system. Social enterprises are certainly not a replacement for government but can work to complement the activities of government at the very local level, where it is often difficult for government agencies to engage appropriately.^3^ Secondly, the work provided by WISEs across the country is likely to empower individuals to experience a sense of accomplishment, meaning, and purpose in their work. From a public health perspective and specifically in the Swedish context, more research is needed, using bigger samples and longitudinal methodology, to better identify what types of wellbeing are fostered by SEs in general, and especially by WISEs, which employ an array of socially excluded groups who might easily experience poor health outcomes. If we are serious about addressing the upstream social determinants of health, we need to consider that some of the answers to addressing these are likely to be found at the level of communities. Communities require adequate and tailored support, power, and resources to help themselves far better than hitherto.

Future studies (carried out by public and health researchers) should consider both quantitative and qualitative longitudinal studies where employees working in social enterprises can be followed over time. Such studies could identify what type of work related activities are linked to their perceptions of improved health and wellbeing, as well as which type of wellbeing is affected by these activities, and identify if they are predominantly hedonic (affective experience, presence of positive emotions and life satisfaction) [59] or eudaimonic (quality of life derived from development of person’s potential fulfilment of personally expressive and self-concordant goals) in nature [60]. According to Corden and Millar, longitudinal research is important for the investigation on ‘how people change’ and on ‘how people respond to change’ [61 p529]. Furthermore, research including a larger sample of work integration social enterprises across the country is warranted.

## Conclusion

To our knowledge, this is the first qualitative case study that explores the perceptions of health and wellbeing among employees of any WISE or SE in Sweden. Interviewed employees in this study perceived that working in the WISE brought them a sense of freedom and increased self-esteem because of the possibility to have an income. They were satisfied with their job (e.g., with the quality of and flexibility at work), and they believed that their work benefited the rest of the society. Working in a WISE gave the interviewees a sense of belonging and togetherness through interaction with co-workers and managers, but also an improved quality of life for themselves and their families. These findings, which are the first such findings in the context of Sweden, provide tentative evidence suggesting that working in a WISE may have a positive impact in different ways. Nevertheless, further studies using larger samples are needed and should address, among others, the question as to what type of wellbeing WISEs foster among employees.

## References

[1] European Union Commission (EUC). Social business initiative: creating a favourable climate for social enterprises, key stakeholders in the social economy and innovation. COM. 2011; 682 final.

[2] Woo C, Jung H. The impact of social enterprises on individual wellbeing in South Korea: the moderating roles of social capital in multilevel analysis. Social Indicators Research. 2022; 159: 433–454. DOI: 10.1007/s11205-021-02759-8

[3] Roy M J, Hackett MT. Polanyi’s ‘substantive approach’ to the economy in action? Conceptualising social enterprise as a public health ‘intervention.’ Review of Social Economy. 2017; 75(2): 89–111. DOI: 10.1080/00346764.2016.1171383

[4] Roy MJ, Donaldson C, Baker R, Kay A. Social enterprise: new pathways to health and well-being? Journal of Public Health Policy. 2013; 34(1): 55–68. DOI: 10.1057/jphp.2012.61 23172049

[5] Thomson H, Atkinson R, Petticrew M, Kearns A. Do urban regeneration programmes improve public health and reduce health inequalities? A synthesis of the evidence from UK policy and practice (1980–2004). Journal of Epidemiology & Community Health. 2006; 60(2): 108–115. DOI: 10.1136/jech.2005.038885 16415258 PMC2577369

[6] Roy MJ, Donaldson C, Baker R, Kerr S. The potential of social enterprise to enhance health and well-being: a model and systematic review. Social Science & Medicine. 2014; 123: 182–193. DOI: 10.1016/j.socscimed.2014.07.031 25037852

[7] De Ruysscher C, Claes C, Lee T, et al. A systems approach to social entrepreneurship. VOLUNTAS: International Journal of Voluntary and Non-profit Organizations. 2017; 28(6): 2530–2545. DOI: 10.1007/s11266-016-9704-5

[8] Ferguson KM. Implementing a social enterprise intervention with homeless, street-living youths in Los Angeles. Social Work. 2007; 52(2): 103–112. DOI: 10.1093/sw/52.2.103 17580772

[9] Ferguson KM, Xie, B. Feasibility study of the social enterprise intervention with homeless youth. Research on Social Work Practice. 2008; 18(1): 5–19. DOI: 10.1177/1049731507303535

[10] Ferguson KM, Islam N. Conceptualizing outcomes with street-living young adults: grounded theory approach to evaluating the social enterprise intervention. Qualitative Social Work. 2008; 7(2): 17–237. DOI: 10.1177/1473325008089631

[11] Bertotti M, Harden A, Renton A, Sheridan K. The contribution of a social enterprise to the building of social capital in a disadvantaged urban area of London. Community Development Journal. 2012; 47(2): 168–183. DOI: 10.1093/cdj/bsr020 27746483 PMC5065089

[12] Munoz SA, Farmer J, Winterton R, Barraket JO. The social enterprise as a space of well-being: an exploratory case study. Social Enterprise Journal. 2015; 11(3): 281–302. DOI: 10.1108/SEJ-11-2014-0041

[13] Farmer J, De Cotta T, McKinnon K, et al. Social enterprise and wellbeing in community life. Social Enterprise Journal. 2016; 12(2): 235–254. DOI: 10.1108/SEJ-05-2016-0017

[14] Jang J, Kim TH, Hong H, Yoo CS, Park J. Statistical estimation of the casual effect of social economy on subjective well-being. VOLUNTAS: International Journal of Voluntary and Non-profit Organizations. 2018; 29(3): 511–525. DOI: 10.1007/s11266-017-9935-0

[15] Calò F, Teasdale S, Donaldson C, Roy MJ, Baglioni S. Collaborator or competitor: assessing the evidence supporting the role of social enterprise in health and social care. Public Management Review. 2018; 20(12): 1790–1814. DOI: 10.1080/14719037.2017.1417467

[16] Macaulay B, Mazzei M, Roy MJ, Teasdale S, Donaldson C. Differentiating the effect of social enterprise activities on health. Social Science & Medicine. 2018; 200: 211–217. DOI: 10.1016/j.socscimed.2018.01.042 29421468 PMC5906639

[17] Song J, Fry R, Mizen A, et al. Association between blue and green space availability with mental health and wellbeing. International Journal of Population Data Science. 2018; 3(4): 921–922. DOI: 10.23889/ijpds.v3i4.921

[18] Poveda S, Gill M, Junio DR, Thinyane H, Catan V. Should social enterprises complement or supplement public health provision? Social Enterprise Journal. 2019; 15(4): 496–518. DOI: 10.1108/SEJ-12-2018-0083

[19] Barraket J. Fostering wellbeing of immigrants and refugees? Evaluating the outcomes of work integration social enterprise. In: Denny S, Seddon F, (eds.), Social Enterprise: Accountability and Evaluation around the World. Abingdon, Oxon: Routledge; 2014: 102–120.

[20] Elmes AI. Work integration social enterprise (WISE) effects on economic participation, social inclusion, health and wellbeing: evaluation of Vanguard Laundry Services. Swinborn: Center for Social Impact Suinburne. Faculty of Business and Law; 2020: 1–140.

[21] Krupa T, Sabetti J, Lysaght R. How work integration social enterprises impact the stigma of mental illness. Social Enterprise Journal. 2019; 15(4): 475–494. DOI: 10.1108/SEJ-12-2018-0075

[22] Henderson F, Steiner A, Mazzei M, Docherty C. Social enterprises’ impact on older people’s health and wellbeing: exploring Scottish experiences. Health Promotion International. 2019: daz102. DOI: 10.1093/heapro/daz102 PMC7585484 31598672

[23] Barraket J, Moussa B, Suchowerska R, et al. Improving health equity for young people? The role of social enterprise: final report. Melbourne: Center for Social Impact Swinburne; 2021. DOI: 10.25916/5f604d4e94b31.

[24] McKinnon K, Kennedy M, De Cotta T. Social enterprises and community well-being in regional Australia. Journal of Sociology. 2021: 1–17. DOI: 10.1177/14407833211035839

[25] Farmer J, Kamstra P, Brennan-Horley C, et al. Using micro-geography to understand the realisation of wellbeing: a qualitative GIS study of three social enterprises. Health & Place. 2020: 62. DOI: 10.1016/j.healthplace.2020.102293 32479370

[26] Roy MJ. Towards a ‘wellbeing economy’: what can we learn from social enterprise? In: Gidron B, Domaradzka A, (eds.), The New Social and Impact Economy: An International Perspective, Nonprofit and Civil Society Studies. Cham: Springer International Publishing; 2021: 269–284. DOI: 10.1007/978-3-030-68295-8_13

[27] Suchowerska R, Barraket J, Qian J, et al. An organizational approach to understanding how social enterprises address health inequities: a scoping review. Journal of Social Entrepreneurship. 2020; 11(3): 257–281. DOI: 10.1080/19420676.2019.1640771

[28] Joyce A, Elmes A, Campbell P, et al. The health and well-being impacts of a work integration social enterprise from a systems perspective. Health Promotion International. 2022: daab052. DOI: 10.1093/heapro/daab052 34015101

[29] Bilbijat T, Rendal JS. Exploring eudaimonia through meaningful work narratives within work integration social enterprises. Social Enterprise Journal. 2017; l17(4): 513–126. DOI: 10.1108/SEJ-02-2021-0013

[30] Defourny J, Nyssens M, Adam S. Documenting, theorizing, mapping and testing the plurality of SE models in Western Europe. In: Defourny J, Nyssens M, (eds.), Social enterprise in Western Europe: Theories, models and practice. New York: Routledge; 2021: 1–17. DOI: 10.4324/9780429055140-101

[31] Gawell M, European Union Commission (EUC). Social enterprises and their ecosystems in Europe. Updated country report: Sweden. Luxemburg: Publications Office of the European Union; 2019. Available at http://ec.europa.eu/social/main.jsp?advSearchKey=socenterfiches&mode=advancedSubmit&catId=22.

[32] Gawell M, Sundin E, Tillmar M. Entrepreneurship invited into the (social) welfare arena. In: Andersen LL, Gawell M, Spear R, (eds.), Social Entrepreneurship and Social Enterprises. Nordic Perspectives. New York and London: Routledge; 2016: 215–231.

[33] Wijkström F, Lundström, T. Den ideella sektorn. Organisationerna i det civila samhället, Stockholm: Sober Förlag; 2002.

[34] Esping-Andersen G. The Three Worlds of Welfare Capitalism. Princeton: Princeton University Press; 1990. DOI: 10.1177/095892879100100108

[35] Svedberg G. Bedömningstillfället, mer än en grund för betygsättning. I Per-OlofErixon (red.), Forskningsarbete pågår. Nationella Forskarskolan i Pedagogiskt Arbete (NaPa). Umeå: Umeå universitet (Umeå University); 2005.

[36] Gawell M. Soci(et)al entrepreneurship and different forms of social enterprise. In: Lundström A, Zhou C, von Fridrichs Y, Sundin E, (eds.), Social Entrepreneurship. Leveraging Economic, Political and Cultural Dimensions. Heidelberg, New York and London: Springer; 2014: 23–41. DOI: 10.1007/978-3-319-01396-1_2

[37] Gawell M. Social enterprises in Sweden: intertextual consequences and hidden paradoxes. In: Defourny J, Nyssens M, (eds.), Social Entrepreneurship: Leveraging Economic, Political, and Cultural Entrepreneurship. New York: Routledge; 2021: 218–234. DOI: 10.4324/9780429055140-13-15

[38] Andrews R, Van de Walle S. New public management and citizen’s perceptions of local service efficiency, responsiveness, equity and effectiveness. Public Management Review. 2013; 5: 762–783. DOI: 10.1080/14719037.2012.725757

[39] Wilkinson RG, Marmot MG, (eds.), Social Determinants of Health: The Solid Facts, second ed. Copenhagen: World Health Organization, Regional Office for Europe; 2003.

[40] WHO. Closing the gap in a generation: health equity through action on the social determinants of health. Commission on Social Determinants of Health. Geneva: World Health Organization; 2008. 10.1016/S0140-6736(08)61690-6 18994664

[41] Swedish Government. Swedish Companies Registration Office; 2018. Retrieved from www.bolagsverket.se (Accessed 24 January 2023).

[42] Kvale S, Brinkmann S. Interviews: Learning the craft of qualitative research interviewing. SAGE Publications Inc.; 2009.

[43] Patton MQ. Qualitative research & evaluation methods: integrating theory and practice. SAGE publications Inc.; 2014.

[44] Yin RK. Case study research: design and Methods, fourth ed. Sage, Thousand Oaks; 2009.

[45] SCB (Statistics Sweden). 2021. Available at https://www.scb.se/hitta-statistik/statistik-efter-amne/befolkning/befolkningens-sammansattning/befolkningsstatistik/pong/tabell-och-diagram/kvartals--och-halvarsstatistik--kommun-lan-och-riket/kvartal-2-2021/.

[46] SCB (Statistics Sweden). Norrlandsfondens Konjunkturbarometer [Norrlandsfonden’s Business Tendency Survey]. 2019. Available at https://www.scb.se/contentassets/3e08e3f84db74e0ca1523855586015e1/norrlandsbarometern19.pdf (accessed 30 August 2022).

[47] Health on Equal Terms. Regional Report, 2018. Available at https://www.regiongavleborg.se/globalassets/regional-utveckling/forskning-och-samhallsmedicin/samhallsmedicin/hur_mar_gavleborg/nationell_folkhalsoenkat/presentation-allmant_halsotillstand.pdf (accessed 29 June, 2022).

[48] Björk J. 2019. Sammanställning av handlingsplaner och genomförandeansökningar för regional utveckling av socialt företagande och socialt entreprenörskap [Overview of action plans and implementations for regional development of social enterprise and social entrepreneurship]. Tillväxtverket [Swedish Agency for Economic and Regional Growth] Available at https://tillvaxtverket.se/download/18.3f46dbe516a53b54a2f103f5/1556279085523/Sam (Accessed 31 October 2022).

[49] Graneheim UH, Lundman B. Qualitative content analysis in nursing research: concepts, procedures and measures to achieve trustworthiness. Nurse Educ Today. 2004; 24(2), 105–12. DOI: 10.1016/j.nedt.2003.10.001 14769454

[50] Hsieh H-F, Shannon SE. Three approaches to qualitative content analysis. Qual Health Res. 2005; 15(9): 1277–88. DOI: 10.1177/1049732305276687 16204405

[51] Elo S, Kyngäs H. The qualitative content analysis process. J Adv Nurs. 2008; 62(1): 107–15. DOI: 10.1111/j.1365-2648.2007.04569.x 18352969

[52] Chandler J. A study to explore the impact of working in a social enterprise on employee health and wellbeing in Greater Manchester. Salford: School of health Sciences College of Health and social care. 2016; 1–368 (PhD dissertation).

[53] Gidron B, Hasenfeld Y. Social enterprises: an organizational perspective. New York: Palgrave Macmillan; 2012. DOI: 10.1057/9781137035301

[54] McKinnon K, Kennedy M, Barraket J, De Cotta T. Is being in work good for wellbeing? Work Integration social enterprises in Australia. Australian Geographer. 2020; 51(3): 361–375. DOI: 10.1080/00049182.2020.1781322

[55] Forslund A. Employment outcomes and policies in Sweden during recent decades. Uppsala: Institute for Evaluation of Labour Market and Education Policy (IFAU). 2019. Available at https://www.ifau.se/globalassets/pdf/se/2019/wp-2019-15-employment-outcomes-and-policies-forslund.pdf.

[56] Pudak J, Simlesa D. Is social entrepreneurship better for workers? The influence of work experience in Croatian social cooperatives on perceived well-being. Croatian Sociological Review. 2020; 50(1): 7–30. DOI: 10.5613/rzs.50.1.1

[57] Ho P-Y, Chan K-T. The social impact of work-integration social enterprise in Hongkong. International Social Work. 2010; 53(1): 33–45. DOI: 10.1177/0020872809348950

[58] Efimov I, Lengen JC, Kordsmeyer A-C, Harth V, Mache S. Capturing and analysing the working conditions of employees with disabilities in German social firms using focus groups. BMC Public Health. 2020; 22: 43. DOI: 10.1186/s12889-022-12689-w PMC8886669 35232418

[59] Diener E, Oishi S, Tay L. Advances in subjective well-being research. Nat Hum Behav. 2018; 2: 253–260. DOI: 10.1038/s41562-018-0307-6 30936533

[60] Waterman AS. Reconsidering happiness: a eudaimonist’s perspective. The Journal of Positive Psychology. 2008; 3: 234–252. DOI: 10.1080/17439760802303002

[61] Corden A, Millar J. Qualitative longitudinal research for social policy – introduction to themed section. Social Policy and Society. 2007; 6(4): 529–532. DOI: 10.1017/S1474746407003867

